# The Snake Venom Rhodocytin from *Calloselasma rhodostoma*—A Clinically Important Toxin and a Useful Experimental Tool for Studies of C-Type Lectin-Like Receptor 2 (CLEC-2) 

**DOI:** 10.3390/toxins5040665

**Published:** 2013-04-17

**Authors:** Øyvind Bruserud

**Affiliations:** Department of Heart Disease, Haukeland University Hospital, N-5021 Bergen, Norway; E-Mail: oeyvind.bruserud@helse-bergen.no; Tel.: +47-98425331; Fax: +47-55972950

**Keywords:** C-type lectin-like receptor 2 (CLEC-2), rhodocytin, *Calloselasma Rhodostoma*, platelets, monocytes, endothelium

## Abstract

The snake venom, rhodocytin, from the Malayan viper, *Calloselasma rhodostoma*, and the endogenous podoplanin are identified as ligands for the C-type lectin-like receptor 2 (CLEC-2). The snakebites caused by *Calloselasma rhodostoma* cause a local reaction with swelling, bleeding and eventually necrosis, together with a systemic effect on blood coagulation with distant bleedings that can occur in many different organs. This clinical picture suggests that toxins in the venom have effects on endothelial cells and vessel permeability, extravasation and, possibly, activation of immunocompetent cells, as well as effects on platelets and the coagulation cascade. Based on the available biological studies, it seems likely that ligation of CLEC-2 contributes to local extravasation, inflammation and, possibly, local necrosis, due to microthrombi and ischemia, whereas other toxins may be more important for the distant hemorrhagic complications. However, the venom contains several toxins and both local, as well as distant, symptoms are probably complex reactions that cannot be explained by the effects of rhodocytin and CLEC-2 alone. The *in vivo* reactions to rhodocytin are thus examples of toxin-induced crosstalk between coagulation (platelets), endothelium and inflammation (immunocompetent cells). Very few studies have addressed this crosstalk as a part of the pathogenesis behind local and systemic reactions to *Calloselasma rhodostoma* bites. The author suggests that detailed biological studies based on an up-to-date methodology of local and systemic reactions to *Calloselasma rhodostoma* bites should be used as a hypothesis-generating basis for future functional studies of the CLEC-2 receptor. It will not be possible to study the effects of purified toxins in humans, but the development of animal models (e.g., cutaneous injections of rhodocytin to mimic snakebites) would supplement studies in humans.

## 1. Introduction

The Malayan pit viper, *Calloselasma rhodostoma*, is found in South-East Asia. It attains an average length of 70-80 cm and rarely more than 1 meter. *Calloselasma rhodostoma* produces potent snake venom containing a large number of toxins that target proteins in the vasculature and the coagulation system [1]. One of these toxins is rhodocytin, which is a ligand for the human C-type lectin-like receptor 2 (CLEC-2); this receptor belongs to the group of C-type lectin receptors (CLRs) that form a superfamily of proteins containing conserved C-type lectin binding domains [2]. CLEC-2 is highly expressed on platelets and megakaryocytes and at lower levels on several other myeloid cells; its activation thereby triggers an intracellular signaling pathway resulting in platelet activation, as well as initiation of immune responses [3,4]. 

## 2. The Clinical Presentation of *Calloselasma rhodostoma* Bites

*Calloselasma rhodostoma* is a major cause of snakebite morbidity in Thailand, Cambodia, Laos, Northwest Malaysia and Java [5,6]. As described in a recent review, relatively few studies of the clinical presentation of snake bites have been published, and accurate statistics of the incidence, morbidity and mortality of snakebites throughout the world are not available [7]. However, the effects of *Calloselasma rhodostoma* bites have been described in previous studies [5,6], and a detailed presentation of the clinical characteristics is given in Table 1 [6]. Generally, the clinical presentation correlates with the severity of envenoming [8], and the symptoms will be more severe in individuals with low body weight or comorbidity, if the bite is located to the face or trunk, by exercise after the bite and if the snake clings to the victim for a longer time [7]. A minority of the patients had no symptoms (48 out of 250 patients). Local symptoms were most common (178 out of the 250 patients) [6]. Local swelling and pain then usually start from minutes to several hours after the bite [8]. Skin discoloring, blistering, bleeding and necrosis may also occur. Systemic or distant hemorrhagic effects (*i.e.*, hemoptysis, hematemesis, macroscopic hematuria) were only seen for a minority of 37 patients and was probably due to more severe envenoming, whereas other signs from distant organs were rare [6,9]. Hypotension and shock is only seen for a small minority of patients; the mortality is therefore generally low and mainly caused by severe hemorrhages or secondary bacterial infections [5,6]. 

**Table 1:** The clinical presentation of snakebites caused by *Calloselasma rhodostoma*; 250 verified bites of *Calloselasma rhodostoma* were identified, and the clinical presentation was analyzed [6]. The results are presented as the fraction of patients and a description of the symptoms/signs.

**Local effects of snakebite in all 250 patients**	
48/250	No local swelling, bleeding or other local reaction.
24/250	Negligible reaction with a maximum extent of swelling of <1 cm difference in circumference between the bitten and healthy extremity.
57/250	Mild local swelling, eventually together with local bleeding or blistering, but without necrosis; 1–4 cm difference in circumference between the bitten and the healthy extremity.
94/250	Moderate local reaction with swelling corresponding to a more than 4 cm difference in the circumference between the affected and the healthy extremity; no necrosis.
27/250	Local necrosis; this occurred mainly on bites located on fingers and toes.
**Systemic or distant effects in all 250 patients**	
37/250	General or distant bleeding tendency.
8/250	Hypotension or shock.
**Hemorrhages in the 37 patients with general bleeding tendency **	
29/37	Hemoptysis (this number may be overestimated, because this diagnosis was based on clinical evaluation alone).
21/37	Skin bleeding, usually discoid ecchymoses.
17/37	Gingival bleedings.
3/37	Hematemesis.
1/37	Macroscopic hematuria.
1/37	Intracerebral hemorrhage.

The common reactions at the local site suggest that local inflammation with extravasation is a part of the reaction to the venom. It is not known how the venom causes distant hemorrhages and whether this is due to an effect on the coagulation factor system or circulating platelets. As will be described below, rhodocytin is a venom component that activates platelets; it is not known whether this toxin contributes to the bleeding tendency, because effects of envenoming on peripheral blood platelet counts or *in vivo* platelet functions have not been investigated. One hypothesis could be that rhodocytin activates platelets and, thereby, causes platelet consumption, followed by thrombocytopenia and bleeding. An alternative hypothesis could be bleeding caused by a general alteration of the platelet function, due to rhodocytin. Most of the bleeding complications in the previous study were from the skin or mucous membrane, and this is consistent with a qualitative or quantitative platelet defect [10]. However, since *Calloselasma rhodostoma* venom contains various toxins other than rhodocytin, the clinical symptoms cannot be explained solely based on the effects of rhodocytin/CLEC-2, but are probably also caused by contributions from other toxins to these complex reactions. 

## 3. Rhodocytin Ligation of the CLEC-2 Receptor

### 3.1. Molecular Characterization of Rhodocytin and Its Interaction with the CLEC-2 Receptor

Snake venoms often contain a large number of toxins that target proteins in the vasculature. The venom, rhodocytin, was purified from *Calloselasma rhodostoma* in the 1990s [11,12]. The crystallographic structure of rhodocytin shows that it is made up of two alpha and two beta chains in the form of a tetramer [13]. A disulfide-linked dimer consists of an alpha and a beta chain and two such dimers form a non-disulfide-tetramer [13,14]. The surface of rhodocytin has a negatively charged cleft that provides a suitable docking surface for the predominately positively charged surface region of CLEC-2, and the flexibility of these molecules seems to strengthen the interaction [15].

### 3.2. Expression and Function of the CLEC-2 Receptor in Normal Cells

The CLEC-2 gene is located in the human natural killer gene complex (NKC) on chromosome 12 [2,6]. This complex contains several families of type II transmembrane C-type lectin-like receptors, and CLEC-2 is part of the Dectin-1 cluster, which also includes MICL, CLEC12B, CLEC9A, CLEC-1, Dectin-1 and LOX-1. These receptors recognize a diverse range of structurally unrelated ligands, including molecular patterns in both endogenous and exogenous ligands, as well as receptor-specific ligands (e.g., podoplanin) [16], and the receptors initiate a variety of immunoregulatory, inflammatory and homoeostatic reactions [2,16]. Podoplanin is a mucin-type sialoglycoprotein that is exposed in a variety of cell types, including brain, heart, kidney, lungs, osteoblasts and lymphoid tissue, and it has recently been identified as an endogenous ligand for CLEC-2 [2,16].

By reverse transcriptase mRNA or Northern blot analyses, the CLEC-2 transmembrane receptor transcripts have been identified in bone marrow, circulating myeloid cells (monocytes, dendritic cells and granulocytes), liver and natural killer (NK) cells [17,18]. A systematic analysis of protein expression has revealed that CLEC-2 protein is detected in platelets, megakaryocytes, liver sinusoidal endothelial cells and liver Kupffer cells [19,20]. Studies in mice indicate that CLEC-2 is highly expressed in platelets and megakaryocytes, whereas the levels in other cell types are lower (Table 2). 

As described above, the CLRs are a superfamily of proteins, including a wide range of molecules, some of which are true lectins that bind to carbohydrate ligands. However, many members of this superfamily only share a basic structural scaffold with the true-sugar binding lectins; this is also true for CLEC-2 [15,21]. The structure of CLEC-2 has been resolved, and the basic scaffold is a conserved C-type lectin fold that is held together by disulfide bonds and hydrophobic interactions [15]. A variant of the standard alpha helix loop extends across the surface of the molecule and contains a stretch of 3–10 helices [15]. The cytosolic tail of CLEC-2 contains a novel sequence, YxxL, known as the hemi-immunoreceptor tyrosine-based activatory motif (hemITAM) [21]. This hemITAM is phosphorylated by Src family kinases upon binding of rhodocytin to the extracellular domain [22,23], and this phosphorylation promotes binding of the tyrosine kinase Syk or Zap-70, by their tandem Src-homology 2 (SH2) domains. CLEC-2 is activated as a dimer [2], and ligation causes activation leading to further downstream signaling. 

**Table 2:** Important biological effects of C-type lectin-like receptor 2 (CLEC-2) ligation: direct effects on CLEC-2-expressing cells and indirect effects mediated through podoplanin expression by the target cells. ITAM, immunoreceptor tyrosine-based activation motif;NK, natural killer cells.

Cell	Expression and functional effects of CLEC-2 ligation/activation
**Direct effects on CLEC-2 expressing cells**	
Platelets and megakaryocytes [18,19]	(i) CLEC-2 ligation induces intracellular tyrosine-phosphorylation signaling cascades mediated by Src, Syk, Vav, SLP-76 and PLCγ family members; (ii) There is also an increase in intracellular calcium levels and; (iii) finally, induction of platelet activation. Thus, Syk is a downstream mediator in platelets, neutrophils, monocytes, dendritic cells and endothelial cells (see below).
Neutrophils [20,21,24]	Murine studies indicate that CLEC-2 activation initiates intracellular signaling through Syk and also affects signaling initiated by Toll-like Receptors (TLRs); this TLR effect is then similar to the effects seen in monocytes. CLEC-2 ligation triggers phagocytosis, and this is probably initiated via the cytoplasmic ITAM-like motif of its cytoplasmic tail. Similar to monocytes, CLEC-2 ligation in neutrophils seems to initiate production and release of IL-6, IL-10 and TNF-α.
Monocytes [20,24]	CLEC-2 initiated Syk-coupled signaling is able to modulate TLR-initiated signaling, and proinflammatory responses are thereby altered. Production and release of IL-6, IL-10 and TNF-α is induced.
Dendritic cells [25]	Intracellular signaling initiated by CLEC-2 ligation in dendritic cells involves many of the same mediators as platelets: CLEC-ligation triggers cell migration via downregulation of RhoA activity and myosin light-chain phosphorylation. F-actin-rich protrusions is triggered by Vav signaling and Rac1 activation. This signaling cascade finally results in rearrangement of the actin cytoskeleton, and dendritic cell migration is thereby promoted.
NK cells [17]	Reverse transcriptase-PCR and Northern blot analysis indicate that CLEC-2 is expressed in NK cells, but the functional effects of CLEC-2 ligation have not been examined.
Liver sinusoidal endothelial cells, liver Kupffer cells [20]	CLEC-2 is expressed on liver sinusoidal endothelial cells and Kupffer cells in both mice and humans, but the functional effects of CLEC-2 ligation on these cells have not been studied.
**Indirect effects in podoplanin expressing target cells**	
Endothelial cells and vessel formation [26,27,28]	Interaction between CLEC-2 in platelets and podoplanin in lymph endothelial cells are necessary for the embryonic separation of lymph and blood vessels; Syk- and SLP-76-deficient mice have blood/lymphatic misconnections. These effects are probably caused by reduced signaling in platelets rather than a direct effect via endothelium-expressed CLEC-2.
Cancer cells and development of metastases [29,30,31,32]	Podoplanin is expressed in several malignancies and seems to be important for cancer cell migration and metastasis. The likely mechanism is cancer-induced platelet activation with the release of soluble mediators that affect endothelial cells and/or cancer cell migration with the development of metastases.

Recent studies suggest that CLEC-2-initiated signaling via Syk in myeloid cells alters signaling initiated by Toll-like receptors (TLRs) and, thereby, modulates inflammatory responses [3], although the details of these interactions are not fully understood. 

### 3.3. The Possible Role of CLEC-2 in Cancer Development

CLEC-2 has a possible role in tumor growth and metastasis [29,30,31,32]. Tumor cell-induced platelet activation seems to be mediated through the release of soluble mediators (adenosine phosphate, thromboxane), and this effect can be further strengthened by the activation of serine proteases (thrombin, capsin B) generated by the procoagulant activity of some tumor cells through (i) the release of matrix metalloproteases from cancer cells and platelets, and (ii) exposure of subendothelial collagen fibers due to tissue degradation. It is also proposed that platelets stabilize vessel growth (especially through vascular endothelial growth factor (VEGF) release) and, thereby, facilitate development of metastases through a proangiogenic effect [29]. Finally, there are also data supporting that CLEC-2 ligand expression in tumor endothelial cells increases adhesion of malignant cells to the vessel wall, followed by extravasation to new metastatic sites; this seems to involve signaling through small GTPases that regulate the actin cytoskeleton. The crosstalk between platelets and tumor cells causes platelet activation and changes in tumor cell morphology, as well as cell surface molecule expression. This possible role of CLEC-2 in carcinogenesis suggests that CLEC-2 may become a therapeutic target in future cancer therapy [29,30].

### 3.4. Biological Studies of Local and Systemic Effects after Calloselasma Rhodostoma Bites—A Hypothesis-Generating Basis for Future Studies of CLEC-2 Biology?

The *Calloselasma rhodostoma* is a major cause of snakebite morbidity in Southeast Asia. The clinical presentation has been known for a long time, and the local and systemic effects after the bites have been described in previous clinical studies. The swelling and local pain suggest that extravasation and local inflammation is important in the pathogenesis, an observation suggesting that venom toxins have effects on endothelial cells and vessel permeability, as well as local recruitment of immunocompetent cells. One possible explanation for the development of necrosis could be platelet activation with microthrombi and ischemia. The available studies suggest that ligation of the CLEC-2 receptor contributes to all these effects; (i) CLEC-2 ligation seems to affect endothelial cells and vessel formation during embryogenesis, as well as during cancer metastasation [26,27,28,31,32], and direct or indirect effects on the endothelium may contribute to local swelling and extravasation; (ii) activation of immunocompetent cells may be an important local proinflammatory effect; and (iii) platelet activation may cause microthrombi, ischemia and necrosis. Even though the snake venom contains a large number of toxins that target several proteins and other toxins than rhodocytin may be more important for the general effects on the coagulation system and distant bleeding complications, the authors hypothesis is that rhodocytin has an important role in the development of clinical symptoms after *Calloselasma rhodostoma* bites. 

CLEC-2 is a member of a protein superfamily containing conserved C-type lectin-like domains and having diverse functions. CLEC-2 is located on platelets, as well as immunocompetent cells, and receptor ligation leads to intracellular signaling and finally platelet activation. The intracellular signaling downstream to CLEC-2 shows similarities between platelets and immunocompetent cells. Even though the available studies are still few, the present knowledge about the CLEC-2 receptor has contributed to our understanding of hemostasis and links platelet biology to other fields in medicine, such as immunity and cancer. 

Two ligands for CLEC-2 have now been identified: the exogenous and soluble rhodocytin and the endogenous and membrane-bound podoplanin [25,32]. Even though the endogenous ligand has been identified, rhodocytin should still be regarded as a useful experimental tool. Even though different ligands bind to the same receptor, they will not necessarily have similar receptor-binding features and, thereby, induce the same downstream signaling effects. This is true for the Angiopoietin-Tie-2 system where the two ligands, Angiopoietin-1 and -2, bind to the same Tie-2 receptor, but may have different functional effects, depending on the biological context [33,34,35]. Further studies are required to clarify whether or not this may be the case also for rhodocytin and podoplanin. Additional questions that need to be answered by future experimental studies are (i) whether downstream signaling differs between soluble and membrane-bound CLEC-2 ligands and (ii) whether podoplanin exists in biologically active soluble forms similar to several cytokine receptors and adhesion molecules [36,37,38]. The availability of two different ligands may then become important in further studies of ligand-initiated intracellular signaling downstream to CLEC-2.

## 4. Conclusions

Snake venom has already made its way from clinical medicine to experimental studies of CLEC-2 biology. Most studies of patients with *Calloselasma rhodostoma* bites are relatively old; they are mainly descriptive clinical characterizations without additional biological studies [5,6,7]. The author suggests that detailed biological studies in patients with *Calloselasma rhodostoma* bites could be performed and possibly also additional studies in experimental animal models (*i.e.*, local cutaneous injections of rhodocytin) could be used to further verify observations in humans. Our available knowledge suggests that rhodocytin is important, especially for the local reactions to these snakebites, and it possibly also contributes to systemic or distant effects. Such biology could then be used as a hypothesis-generating basis for future functional studies of CLEC-2. The CLEC-2 receptor seems to represent an important link between coagulation, inflammation, immunity and carcinogenesis, and detailed biological studies are therefore important to clarify whether this receptor or its downstream signaling cascade could be considered as a possible therapeutic target in clinical medicine. The downstream signaling from CLEC-2 shows similarities between different cells (*i.e.*, platelets, granulocytes and monocytes; see Table 2) and targeting of CLEC-2 or CLEC-2-induced signaling may therefore represent a possibility to target different proinflammatory cells or different biological processes through a single molecular target.
